# Microwave-Assisted Extraction/UHPLC-Q-Orbitrap-MS-Based Lipidomic Workflow for Comprehensive Study of Lipids in Soft Cheese

**DOI:** 10.3390/foods13071033

**Published:** 2024-03-28

**Authors:** Maria Campaniello, Valeria Nardelli, Rosalia Zianni, Michele Tomaiuolo, Oto Miedico, Marco Iammarino, Annalisa Mentana

**Affiliations:** Istituto Zooprofilattico Sperimentale della Puglia e della Basilicata, Via Manfredonia, 20-71121 Foggia, Italy; maria.campaniello@izspb.it (M.C.); valeria.nardelli@izspb.it (V.N.); michele.tomaiuolo@izspb.it (M.T.); oto.miedico@izspb.it (O.M.); marco.iammarino@izspb.it (M.I.); annalisa.mentana@izspb.it (A.M.)

**Keywords:** Microwave-Assisted Extraction, foodomics, lipidomics, UHPLC-Q-Orbitrap-MS, experimental design, chemometric analysis, soft cheese

## Abstract

In this work, Microwave-Assisted Extraction (MAE) was proposed as an alternative and environmentally friendly technique in lipidomics to study the lipid fingerprint of soft cheeses, such as mozzarella. For method development, a first step concerning an evaluation of extraction solvents was carried out via testing three different mixtures, including methanol/ethyl acetate, isopropanol/ethyl acetate, and ethanol/ethyl acetate, at a 1:2 *v*/*v* ratio. The latter was chosen as a solvent mixture for subsequent method optimization. MAE conditions, in terms of solvent volume, time, and temperature, were explored to define their effects on extraction capability through a full factorial experimental design. The best compromise to extract more lipids at the same time was obtained with 24 mL g^−1^ for solvent-to-solid ratio, 65 °C for temperature, and 18 min for time. Lipid analyses were conducted by UHPLC-Q-Orbitrap-MS associated with multivariate statistics. The developed lipidomic workflow allowed for the extraction of over 400 lipids grouped into 18 different subclasses. The results confirmed that MAE is a suitable technique for lipid extraction in the omics approach with high efficiency, even using low-cost and less toxic solvents. Moreover, a comprehensive structure characterization of extracted lipids, in terms of fatty acid composition and regiochemistry, was carried out.

## 1. Introduction

Traditional lipid analysis in the food field, such as total fat content, fatty acid composition, and physico-chemical analysis, can only provide basic information (the content of lipid class and oxidative state), while little information is given regarding the composition of these molecules and their structures [1]. Lipidomics focuses on analyzing the complete lipid profile of a biological and food system. It is a specialized field within metabolomics, and by means of powerful analytical technologies associated with multivariate statistics, it provides fast, comprehensive, and precise insights for descriptive and discriminative analyses of lipids [2]. The purposes of lipidomics can be many and different, such as the characterization of nutritionally important lipids, the study of food authentication, adulteration, and safety assurance [1]. Moreover, through the untargeted approach, it is possible to identify potential new authentication and technological markers, which are also useful in traceability and food safety control plans [3].

Generally, a complete MS-based lipidomics workflow involves sample treatment, analysis by means of mass spectrometry, data processing using dedicated software for the identification of compounds, and elaboration and interpretation of data by appropriate statistical tools. In such an organized workflow, sample treatment, more specifically the extraction, represents the first step to isolate a subset of components and/or to remove some interferents. Hence, this step is fundamental for high-quality and exhaustive analysis [4]. Lipidomic studies often require optimizing more than one extraction due to the chemical and structural diversity among lipid classes and subclasses [5]. The selection of appropriate solvents or solvent mixtures and the optimization of extraction settings, such as temperature, time, and solvent amount, are critical factors for efficient lipid extraction; thus, several methods have been developed using organic solvents with different polarity and experimental conditions. Specifically, Folch [6] and Bligh and Dyer [7] developed two procedures for the extraction of polar and non-polar lipids from food samples, also applicable to milk and cheeses. Moreover, these methods can be associated with sample pre-cleaning or pre-fractionation by solid phase extraction columns or thin-layer chromatography prior to LC-MS analysis to facilitate lipid species identification and improve the quality of analytical data [8]. On the other hand, these two classic lipid extractions are characterized by large use of solvents and long-time analysis.

According to the recent trend in analytical chemistry beyond green chemistry, alternative environmentally friendly techniques or solvents were considered for lipid extraction. Matyash et al. [9] introduced methyl-tert-butyl ether (MTBE) as a non-toxic solvent for the extraction of lipids from biological samples; this solvent was also associated with other techniques, such as ultrasound extraction [10]. Recently, Accelerated Solvent Extraction (ASE) was proposed as an alternative and efficient solvent extraction technique for lipid analysis [11]; this automated technique, through the use of high temperature and pressure, allows for reducing the quantity of organic solvent necessary for extraction and the process times [12].

Microwave-Assisted Extraction (MAE) is an extraction technique used for different scientific applications that combines the action of microwave energy with solvent extraction capability [13]. This technique is characterized by low usage of solvents with consequently less laboratory waste along with the ability to automate the extraction process reducing process times and sample preparation costs, as well as improving extraction efficiency [13,14]. This technique consists of heating the extractant (mostly liquid organic solvents) in contact with the sample by means of microwave energy. The partitioning of the analytes of interest from the sample matrix to the extractant depends on its temperature and physical–chemical properties [15]. 

MAE in food analysis was useful for the extraction and determination of labile flavor compounds [16] or active molecules, successively used in the food, cosmetic, and pharmaceutical industries [17,18]. Moreover, MAE was used for total and healthy lipid recoveries and for essential oils, evaluating total fats, fatty acid composition, and volatile compounds together with other indices as cytotoxic, antioxidant, anti-inflammatory, or antimicrobial properties in different matrices [19,20,21,22,23]. Furthermore, Liu et al. compared conventional and green extraction methods on oil yield, physicochemical properties, and lipid compositions, specifically fatty acids, triacylglycerols, and lipid concomitants (squalene, β-sitosterol, and tocopherols) in pomegranate seed oil [24]. Gómez-Brandón et al. used MAE for determining phospholipid fatty acids in solid environmental samples [25]. Medina et al. developed a precise, accurate, and rapid MAE method for lipids in different meats by evaluating fatty acids and lipid oxidation products [13].

Soft cheeses are relevant dietary components for human health because they contain both the major macronutrients (carbohydrates, lipids, proteins) and minor constituents (vitamins, minerals, etc.) [26]. In particular, the demand for mozzarella is increasing worldwide due to its sweet and fresh taste, which is appreciated by consumers. The deepening of the chemical composition of dairy products can enrich the knowledge about nutritional quality and safety; i.e., polar lipids, such as phospholipids and sphingolipids, are indispensable essential constituents of cell membranes and are involved in regulating cell signaling. Moreover, these lipids have a healthy effect on infant and adult cognitive processes and on cardiovascular functionality [27]. Furthermore, some specific lipids may be used for the authentication and valorization of typical dairy products [26].

Studies on MAE applied to milk and cheeses were carried out for the extraction and determination of aflatoxins and pesticides [28,29], biogenic amines [30], bioactive peptides [31], and organic acids [32]. Moreover, Dvoršćak et al. described for the first time the use of MAE for organic pollutants from human milk samples, highlighting the influence of sample preparation and pretreatment [33]. Pombal et al. developed a simple and rapid method for fast quantitation of total fat in cheese samples using a new method based on an innovative MAE [34]. González-Arrojo et al. used MAE, combining extraction and derivatization steps into a single one for the determination of FAs in milk [35].

In our previous research, untargeted lipid fingerprints of soft cheeses were studied by successfully applying Folch and ASE extraction techniques [11] and evaluating the effect of technological processes on the lipid composition of cheese [3]. However, to our knowledge, no studies using MAE associated with untargeted lipidomics have been reported.

For these reasons, in this study, an MAE combined with Ultra High-Performance Liquid Chromatography–Orbitrap Mass Spectrometry (UHPLC-Q-Orbitrap-MS) analysis for the lipid fingerprint of mozzarella cheese was developed. The experimental design was based on a first screening step to test three solvent mixtures (methanol/ethyl acetate, ethanol/ethyl acetate, IPA/ethyl acetate). Successively, the best solvent mixture was used in a full factorial design (FFD) with two levels and a central point, and a Pareto front was built to investigate the effects of three independent variables (time, temperature, and solvent-to-solid ratio) to achieve the final optimal MAE settings for the extraction of lipids from mozzarella samples. Moreover, in this work, a comprehensive structure characterization of extracted lipids in terms of fatty acid composition and regiochemistry was carried out.

## 2. Materials and Methods

### 2.1. Chemicals and Reagents

Sodium sulfate (Na_2_SO_4_) analytical grade, ammonium formate (NH_4_HCO_2_), isopropanol (IPA), water (H_2_O), acetonitrile (ACN), and formic acid (HCO_2_H) of LC/MS grade, were provided by Carlo Erba Reagents (Cornaredo, MI, Italy). Ethyl acetate analytical grade was purchased from Fluka (Buchs, Switzerland). Chloroform (CHCl_3_) HPLC grade, ethanol, and methanol (MeOH) of LC/MS grade were provided by Merck Life Science S.r.l. (Darmstadt, Germany) and EMD Chemicals (Gibbstown, NJ, USA). Pierce LTQ Velos ESI Positive and Negative Ion Calibration Solutions were provided by Thermo Fisher Scientific (Waltham, MA, USA). The 1,2,3-tripelargonoyl-glycerol (trinonanoin, 9:0-9:0-9:0-TAG) and the deuterated lipid internal standards, Equisplash™ Lipidomix^®^ 100 mg L^−1^, were purchased from Merck Life Science S.r.l. (Darmstadt, Germany). Stock standard solution and working standard solution of trinonanoin, 10,000 mg L^−1^ in CHCl_3_/MeOH (1:1, *v*/*v*) and 1000 mg L^−1^ in MeOH/CHCl_3_ (4:1, *v*/*v*), respectively, were prepared and stored at 4 °C (±2 °C).

### 2.2. Microwave-Assisted Extraction (MAE) Procedures

All mozzarella cheese samples were purchased from local markets and stored at 4 °C (±2 °C). MAE was performed using an ETHOS-ONE microwave system with a 100 mL Teflon vessel (Milestone s.r.l., Sorisole, Bergamo, Italy). For the first step of extraction solvent evaluation, 12 mL of each solvent mixture, namely, methanol/ethylacetate (ME), ethanol/ethyl acetate (EE), IPA/ethyl acetate (IE) in a ratio of 1:2 *v*/*v* was tested and individually added to 0.5 g of homogenized mozzarella sample. The working standard solution of trinonanoin was spiked as the internal standard (200 μL). A microwave temperature program was set to heat the sample to 65 °C for 15 min; then, the temperature was held for 18 min and finally reduced to 25 °C. The maximum extraction power was set to 700 W. This setting corresponds to the values of the central point belonging to the FFD constructed for the optimization of the extraction procedure (Table 1 and Table 2). The extract was collected in a 50 mL falcon and evaporated to dryness at 40 °C under nitrogen flow using an automated solvent evaporation system TurboVap^®^ II (Biotage AB, Uppsala, Sweden). All dried extracts were suspended in MeOH/CHCl_3_ (4:1, *v*/*v*) to obtain a final concentration of 2000 mg L^−1^, centrifuged at 400 RCF for 10 min at 4 °C (±2 °C), and then the supernatant was analyzed by UHPLC-Q-Orbitrap-MS.

### 2.3. Folch Procedure

Folch method was carried out on 0.5 g of homogenized mozzarella cheese spiked with 200 μL of working standard solution of trinonanoin as internal standard. The extraction procedure was performed according to our previous article [11].

### 2.4. Untargeted Analysis

All analyses were performed using an Ultimate 3000 UHPLC coupled with a Q-Exactive Focus Orbitrap Mass Spectrometer (Thermo Fisher Scientific, Waltham, MA, USA) equipped with a heated electrospray ionization (HESI) source. The chromatographic conditions and the analytical parameters are shown in Table S1. In this study, a procedural blank, defined as Quality Assurance (QA), was used to ensure the performance and final results of the experiments. It was also used for search and alignment during Lipidsearch^TM^ elaboration. Quality control (QC) was carried out by adding a mixture of Equisplash™ Lipidomix^®^ and trinonanoin to the sample matrix, which was injected every 10 runs. The RSD% of the deuterated lipids in QC samples was lower than 20%, confirming the stability and reproducibility of chromatographic runs and MS acquisitions (Table S2) [36]. Finally, a Pooled Sample (PS) prepared by mixing equal 150 µL aliquots of six lipid extracts was injected at the beginning of the analytical batch for conditioning the chromatographic system [3]. Successively, UHPLC-Q-Orbitrap-MS data were processed by LipidSearch^TM^ v4.2.2.7 software (Thermo Fisher Scientific, Waltham, MA, USA) [37]. Data elaboration and lipid identification consisted of two phases: *search* and *alignment*. During the *search*, the software compared each “data-dependent” mass spectrum with the proprietary library containing thousands of lipids mass spectra. Each peak was classified with a score resulting from overlap with the theoretical spectra, providing a highly accurate identification of the fatty acid composition and sn1, sn2, or sn3 position [11]. Detailed software parameters are reported in Table S1. For oxidized lipids, “Oxid. GPL” was inserted in the Lipidsearch^TM^ database, and their identification was also supported using FreeStyle^TM^ v1.6 software (Thermo Fisher Scientific, Waltham, MA, USA) [3].

### 2.5. Experimental Design and Data Analysis

The experimental design and all statistical and chemometric analyses were performed using free software R version 4.1.1 [38] using in-house routines, partly based on the mdatools package [39].

## 3. Results and Discussion

### 3.1. Solvent Mixture Selection

Considering that lipid isolation follows the laws of solid–liquid extraction, it is common to perform MAE using the same solvent prescribed for traditional extraction, such as Folch [6] or Bligh and Dyer [7]. However, the optimal solvents for MAE cannot always be deduced directly from those used in conventional procedures [15], so an opportune evaluation of solvents is necessary. Moreover, according to the recent trend in analytical chemistry beyond green chemistry, in this study, alternative environmentally friendly solvents were considered. Taking into account the dielectric characteristics, it seemed reasonable to use ethyl acetate, polar aprotic solvent, instead of chloroform (dielectric constant ε′: 6.8 and 4.8, respectively) while, as an alcoholic polar component, methanol, used for traditional extraction, was compared to IPA and ethanol. Therefore, the mixtures methanol/ethyl acetate (ME), ethanol/ethyl acetate (EE), and IPA/ethyl acetate (IE), at a ratio of 1:2 *v*/*v*, were tested.

Eighteen lipid subclasses, including also oxidized forms, were identified. Specifically, in positive ion mode, 322 triacylglycerols (TG) and 24 related oxidized forms with one or two oxygens on fatty acid chains (TG_1OX and TG_2OX) were identified as +NH_4_ or +Na and +NH_4_, +Na, or +H adducts, respectively, and distinguished by the composition of fatty acids and positional isomers. Moreover, 39 diacylglycerols (DG) and 1 related oxidized form (DG_OX) as +NH_4_ adduct, 8 lysophosphatidylcholines (LPCs), 3 phosphatidylcholines (PC), 8 phosphatidylethanolamines (PE) as +H adduct, 1 bis methyl phosphatidic acid (BisMePA) as +Na adduct and cholesterol ester (ChE) as +H-H_2_O adduct were measured. In negative ion mode, six ceramides (Cer), seven hexosyl ceramides (4 Hex1Cer and 3 Hex2Cer), six phosphatidylcholines (PC), and eight sphingomyelins (SM), all as +HCOO adducts, together with five phosphatidylinositols (PI), three phosphatidylserines (PS), four lysophosphatidylethanolamines (LPE), two lysophosphatidylserines (LPS), and one lysophosphatidylinositol (LPI) as -H adducts, were identified.

**Figure 1:**
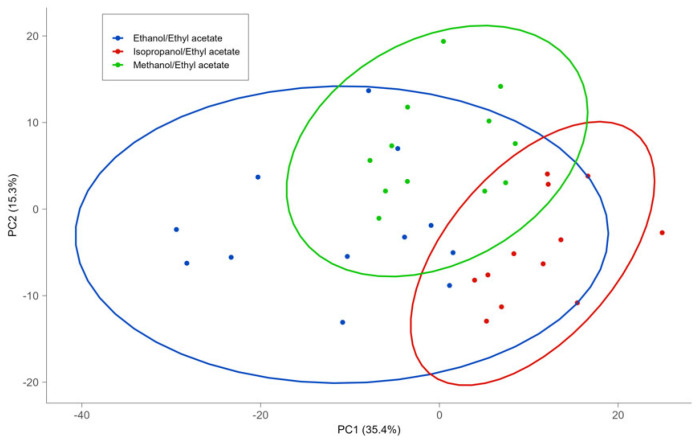
Score plots of PCA of lipids extracted using different solvent mixtures.

**Figure 2:**
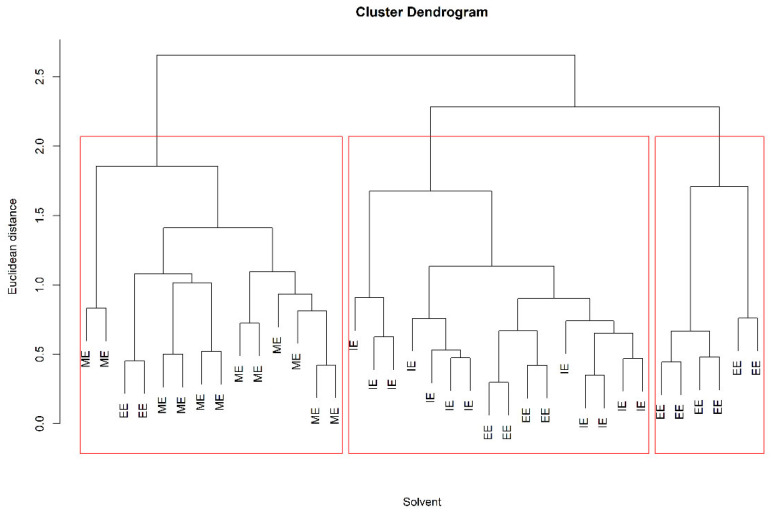
Dendrogram of hierarchical cluster analysis (HCA) of different solvent mixtures. Methanol/ethyl acetate (ME), ethanol/ethyl acetate (EE), and IPA/ethyl acetate (IE), all mixtures were at a 1:2 *v*/*v* ratio.

The results showed that there was no variation in the qualitative lipid fingerprint (in number and type) of mozzarella for the three solvent mixtures tested (Figures S1 and S2). On the other hand, the yield of lipid extraction using ME and EE was higher than 85% and higher than the yield of IE (around 75%). Moreover, differences in the abundance of specific lipids were observed and then considered for statistical analysis. In particular, the evaluation and discrimination of extractions with different solvent mixtures were verified using Principal Component Analysis (PCA), Hierarchical Clustering Analysis (HCA) (Figure 1 and Figure 2), and Partial Least Squares–Discriminant Analysis (PLS-DA) (Table 3). The PLS-DA model quality assessment was carried out in a double cross-validation schema, while predicted uncertainties were also estimated by means of bootstrap.

The score graph obtained from the PCA (Figure 1) shows no clustering among the solvents. A partial grouping is evident with the dendrogram obtained from HCA (Figure 2), in which the sum of lipids in the different subclasses was used as a variable for the cluster search. These variables were subjected to a pretreatment consisting of scaling to the range [0, 1], and the dendrogram was obtained by implementing the squared Euclidean distance and the average linkage method. In the dendrogram corresponding at a value of Euclidean distance of two, three clusters were obtained. The first group that was formed, highlighted on the right of Figure 2, consists of all EE extractions, while the central cluster contains 12 IE extractions and 4 EE extractions. Finally, the third cluster, at the left of Figure 2, contains 12 extractions with ME and only 2 extractions with EE. The HCA provided a clearer separation between the ME and IE than the EE mixture. On the other hand, the models obtained by PLS-DA (Table 3) allowed us to classify the ME and IE with excellent values of diagnostic statistics, whereas EE extractions are not clearly discriminated against with respect to the other extractions.

Summarizing, no qualitative difference was observed in the lipid fingerprint of mozzarella samples using the three alcohols, and furthermore, the variation in extraction capability of ME for the individual lipid subclasses compared to EE was lower than 20% (Figure S3) with a similar total fat recovery. In conclusion, considering that the aim of this study was the development of a more environmentally friendly MAE method, ethanol was chosen as the alcoholic component for further experiments. It combines lower toxicity and cost with respect to methanol, which is a toxic solvent with negative effects on the environment.

### 3.2. Full Factorial Design

In this study, a 2^k^ factorial design (FFD) with three experiments for the central point was employed to evaluate the most relevant factors in lipid extraction by MAE. Three experimental factors, solvents-to-solid ratio (X_1_, mL g^−1^), extraction time (X_2_, min), and temperature (X_3_, °C), were screened using two levels, coded as −1 and +1 for their effects on dependent variables, namely, the sums of lipids divided into 18 subclasses. For our experimental design, X_1_ was selected from 16 mL g^−1^ to 32 mL g^−1^. The ranges of other independent parameters were 6 min–30 min for X_2_ and 50 °C–80 °C for X_3_ (Table 1). Eight experimental conditions obtained from the FFD were studied. The addition of the central point analyzed in triplicate was dictated by the need to better describe the possible effects due to the interactions between the three variables, increasing the overall dataset to 11 runs (Table 2).

The mathematical model is, therefore, the following:

$$Y=b_{0}+\left(\sum_{i=1}^{3}b_{i}X_{i}\right)+\left(\sum_{i=1}^{2}\sum_{j=i+1}^{3}b_{ij}X_{ij}\right)+b_{123}X_{1}X_{2}X_{3}$$

The obtained models showed good descriptive capacity of the data, with the *p*-value of the models from 0.049 for TG_2OX to 2.43 × 10^−5^ for LPI and all R^2^adj being very good (Table 4). More specifically, the significant coefficients obtained with the elaboration suggest that LPC, LPE, LPI, and LPS extractions were improved proportionally to the solvent-to-solid ratio (X_1_) increasing, while the extracted amounts of Cer, PC, PE, and PI showed an opposite trend. On the other hand, the extraction time (X_2_) negatively affected Cer, Hex1Cer, PC, PE, and PI extractions. Finally, the temperature increase (X_3_) favored LPC, LPI, and LPS extraction. Regarding the interaction among those factors, the solvent-to-solid ratio never interacted with the time (X_1_ X_2_) except for LPI and PC, which produced a relatively low coefficient for X_1_ X_2_ interaction. Only temperature interacted more with the other two variables (X_1_ X_3_ and X_2_ X_3_). When the temperature (X_3_) influences the extraction with its linear component, this variable also appears in interaction with the solvent-to-solid ratio. This is the case for LPC, LPI, LPS, and PC. For Cer, Hex1Cer, PC, and PE subclasses, the time affected in the linear term (X_2_) is also observed as a time–temperature interaction (X_2_ X_3_). Finally, the significant coefficients obtained suggested that X_1_ positively affected TG_2OX.

The lipid subclasses not reported in Table 4 did not show any significant dependence related to investigated factors.

In this study, more than one response was studied, meaning that, unless interested in specific lipid subclasses, looking at all subclasses at the same time was needed to find the best compromise. Therefore, the PCA and Pareto front were elaborated. For this elaboration, a single dataset containing all the lipid subclasses that showed a certain dependence on the experimental conditions in the regression models (from positive and negative datasets) was used.

**Figure 3:**
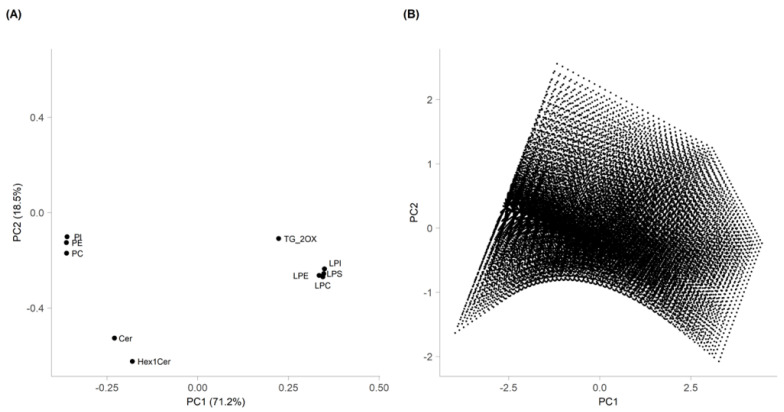
Loading plot of PCA obtained from FFD elaboration (**A**) and Pareto front (**B**), estimated with the regression models.

In Figure 3A, the first two main components of PCA, PC1 and PC2, accounted for 71.2% and 18.5% of the total variance, respectively. In order to obtain the Pareto front, a linear model with interactions was created for PC1 and PC2, considering the values of X_1_, X_2_, and X_3_ as the independent variables of the experimental design. Subsequently, 40 equally distributed values were defined for each variable in the experimental range, and a grid of the possible combinations of 40 values (64,000 points) was constructed. The values of PC1 and PC2 were calculated for each set of values of the grid on the basis of the two models. The resulting pairs of values represented the coordinates of points in the PC1-PC2 plane.

The study of the loading plot (Figure 3A) and Pareto front (Figure 3B) showed that the optimal extraction conditions depended on the lipid subclasses.

More specifically, for TG_2OX, LPC, LPE, LPI, and LPS, the highest recovery was obtained toward the maximum of PC1, which corresponded to the maximum solvent-to-solid ratio, temperature, and extraction time. For PI, PE, PC, Cer, and Hex1Cer, the best extraction conditions were obtained toward the minimum of PC1, which corresponded to the minimum solvent-to-solid ratio, temperature, and extraction time. Definitely, the best compromise to extract all the lipids at the same time corresponded to the conditions of the central point.

### 3.3. Lipid Characterization

An in-depth characterization of the fatty acid composition of lipids was performed in this section. Triacylglycerols were the most abundant lipids, characterized by three positions on the glycerol backbone: sn-1; sn-2; and sn-3. In the sn-1 position, out of 322 TG, 274 were saturated fatty acids (SFA), 48 were monounsaturated (MUFA), and no polyunsaturated (PUFA) were found. Regarding the number of carbon atoms of fatty acids (FA), about 46% were short-chain (S-C; C 4–10); 10% were medium-chain (M-C; C 11–15), and 44% were long-chain (L-C; C > 16). Butyric acid (4:0) was the main applicant in 27% of TGs.

In the sn-2 position, in addition to 205 SFA and 101 MUFA, 16 PUFAs were present, mainly linoleic acid (18:2). In a similar way to what is described for sn-1, S-C and L-C FA were predominant, with 34% and 46%, respectively. The most abundant FA in position sn-2 was oleic acid (18:1), which was present in 60 TG. The sn-3 position was characterized by an equal distribution in SFA, MUFA, and PUFA; the most frequent were myristic (14:0), palmitic (16:0), oleic (18:1), linoleic (18:2), and α-linolenic (18:3) acids. It was noted that in position sn-3, L-C and FA were found in 80%, while S-C and M-C were found in 5% and 15%, respectively. Furthermore, the presence in this position of arachidonic acid (20:4), eicosapentaenoic acid (20:5), and docosapentaenoic acid (22:5) was underlined. These L-C PUFAs can reduce oxidative stress and may have potentially beneficial effects on cardiovascular disease and neurological disorders [40].

In general, TGs were also characterized by the presence of saturated odd chain fatty acids (OCFA), pentadecanoic acid (15:0), and heptadecanoic acid (17:0). Humans cannot synthesize 15:0 and 17:0 FA, so dairy products can be considered as primary dietary sources. The incorporation of 15:0 and 17:0 into cell membranes can enhance membrane fluidity, and some studies have linked these OCFAs to a reduced risk of coronary heart disease and diabetes [41].

Similarly, DGs were also characterized by the position of the fatty acid chain on the glycerol backbone. The two isomers, sn-1,2-DG and sn-2,3-DG, were indistinguishable in untargeted lipidomic, so they were named sn-1 and sn-2 as a convention. In DG, similar to TG, the sn-1 location showed more abundant FA distribution in SFA (87%), while 13% were MUFAs, and no PUFAs were found. Once again, chain length corresponds to the TG sn-1 trend: 44% S-C; 10% M-C; 46% L-C. The sn-2 location was characterized by 17 SFAs, 10 MUFAs, and 12 PUFAs. L-C FAs were 64%, and the more abundant were oleic and linolenic acids; M-C was 28%, with the main presence of myristic acid.

Thirteen TG_1OX, specifically nine with the presence of 18:1+O and four with 18:2+O, 11 TG_2OX and 1 DG_OX with double oxidized linoleic fatty acid (18:2+OO) were identified. Six of these were also detected in their non-oxidized form. All oxidized lipids were identified as grades “A” or “B”. The study of oxidized species is an emerging branch of lipidomics, which lacks guidelines and, therefore, only the most confident identifications, i.e., the presence of oxidized neutral loss and grades “A” or “B” with the support of manual evaluation of MS/MS spectra, were retained [3].

The minor constituents of the lipidome were Cer, HexCer, SM, PC, PE, PI, PS, and their respective lyso forms. The structures of Cer and HexCer were characterized by a sphingosine (d18:1) or hexa-deca-4-sphingenine (d16:1) backbone, with the addition of a long chain fatty acid to the amine group and a sugar linked to OH for HexCer. L-C SFA 16:0, 22:0, 23:0, and 24:0 were detected, as was also found in bovine milk by Liu et al. [42].

The phospholipids PC, PE, PI, and PS showed a predominance of 16:0, 18:0, 18:1 in sn-1 and 18:1 and 18:2 in sn-2. Sphingomyelins showed a total number of carbons ranging from 32:1 to 42:1 and one unsaturation, except 39:0. Lysophospholipids showed the same FA as phospholipids and, in addition, 12:0 and 15:0 were also found. Details on the individual lipids identified are shown in Supplementary Data (Table S3).

### 3.4. Comparison of MAE and Folch Methods

**Figure 4:**
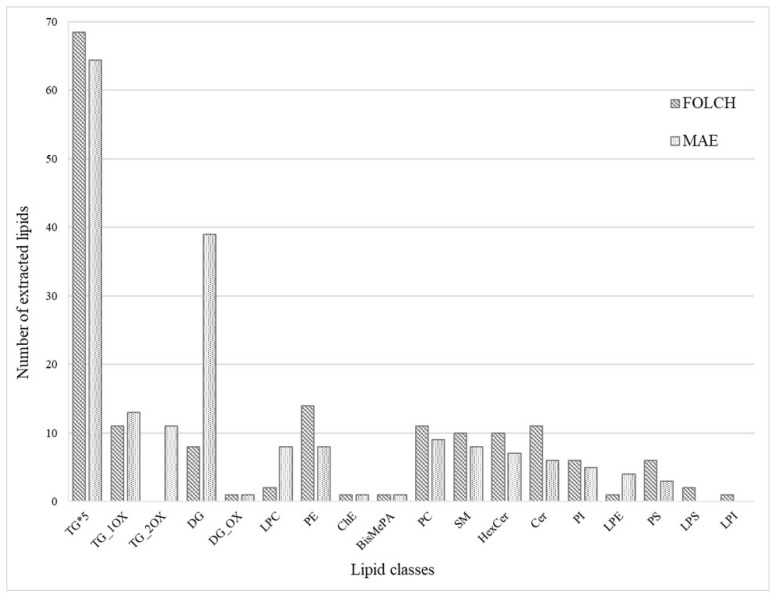
Comparison of the number of lipids extracted from mozzarella cheese between MAE and Folch procedures.

The lipid profile obtained from mozzarella cheese by the optimized MAE method was compared with that of the Folch procedure, and some differences were highlighted. Folch extraction allowed for a higher number of TG and other polar lipids, i.e., PE, PC, SM, Cer, HexCer, PI, and PS (Figure 4). Moreover, LPS and LPI were only extracted using the Folch procedure. These results can be explained by the use of a large volume of methanol in Folch, which allowed for a more efficient breaking of the bonds between lipids and biopolymers [43] and permitted the extraction of the membrane lipids [44] On the other hand, MAE extracted more TG_OX, DG, LPC, and LPE compared to Folch’s procedure. Regarding lysophospholipids, it is reasonable to state that MAEs do not produce these molecules, but optimized extraction conditions can favor their extraction since, as is known from the literature, they are milk components [42]. Differently, oxidized triglycerides, in particular TG with double oxygen, can be linked to the in situ formation by means of microwaves during MAE extraction, but it cannot be excluded that they are milk components because similar compounds were already detected in raw milk [45].

## 4. Conclusions

In this work, a lipidomic workflow based on MAE/UHPLC-Q-Orbitrap-MS was developed for mozzarella cheese. The extraction solvent screening showed no differences in terms of qualitative lipid fingerprints among the tested mixtures, so eco-friendly ethanol/ethyl acetate was used for the subsequent extraction optimization by FFD. The results highlighted that the extraction parameters, especially the solvent-to-solid ratio, had a high impact on the extracted amount of some lipids, above all phospholipids and ceramides, which are important lipid subclasses to be considered in lipidomic studies. This optimized approach allowed for the identification of over 400 lipids, grouped into 18 different subclasses, i.e., TG and their oxidized forms, DG and their oxidized forms, ChE, BisMePA, Cer, HexCer, SM, PC, PE, PI, PS, LPC, LPE, LPI, and LPS.

Considering the complexity of the lipidomic workflow, the possibility of reduced usage of solvents with consequently less laboratory waste, along with the ability to automate the extraction process, is an important result. The outcomes confirmed the adaptability of this optimized MAE for the lipidomic approach in foodomics, and the use of eco-friendly solvents increased the green aspect of this technique, proving itself as a less toxic and more efficient alternative to the Folch method. This approach can be useful in different applications, such as food authentication, and as a model for food quality and safety control.

## Figures and Tables

**Table 1:** Coded and real value assignments for FFD and experimental matrix.

Factors	Factor Levels		
	−1	0	+1
Solvent-to-solid (mL g^−1^)	16	24	32
Time (min)	6	18	30
Temperature (°C)	50	65	80

**Table 2:** Experimental matrix obtained from FFD.

Run	Solvent-to-Solid (mL g^−1^) X_1_	Time (min) X_2_	Temperature (°C) X_3_
1	16	6	50
2	32	6	80
3	16	30	80
4	32	6	50
5	32	30	80
6	16	6	80
7	16	30	50
8	32	30	50
Central	24	18	65
Central	24	18	65
Central	24	18	65

**Table 3:** Diagnostic statistics for PLS-DA model.

Double Cross Validation				Bootstrap		
Parameter	Ethanol/Ethyl Acetate	IPA/Ethyl Acetate	Methanol/Ethyl Acetate	Ethanol/Ethyl Acetate	IPA/Ethyl Acetate	Methanol/Ethyl Acetate
RMSECV	0.572	0.397	0.371	0.620	0.421	0.430
Q2	0.631	0.822	0.845	0.569	0.800	0.792
DQ2	0.692	0.867	0.860	0.633	0.844	0.809
Accuracy	0.933	0.998	1.000	0.916	0.993	0.986
Sensitivity	0.810	1.000	1.000	0.778	0.998	0.978
Specificity	0.994	0.997	1.000	0.986	0.990	0.990

**Table 4:** Significant equation coefficients of the models obtained from FFD elaboration.

	Cer	Hex1Cer	LPC	LPE	LPI	LPS	PC	PE	PI	TG_2OX
**β_0_**	0.474	0.453	0.335	0.428	0.364	0.343	0.502	0.512	0.425	0.115
**β_1_**	−0.119		0.334	0.344	0.345	0.331	−0.241	−0.238	−0.286	0.141
**β_2_**	−0.112	−0.203			0.021		−0.114	−0.115	−0.131	
**β_3_**			0.117		0.096	0.109	−0.065	−0.071		0.137
**β_12_**					0.034		−0.061			
**β_13_**			0.115		0.096	0.121	−0.066			
**β_23_**	0.236	0.166			−0.072		0.115	0.090		
**β_123_**		−0.212			−0.081	−0.075	−0.080	−0.076		
**R^2^adj**	0.897	0.857	0.967	0.921	0.999	0.972	0.97	0.948	0.897	0.848
***p*-value of model**	0.028	0.045	0.005	0.019	2.43 × 10^−5^	0.004	0.005	0.010	0.028	0.049

Significant coefficients are in grey when *p* < 0.05 and in black when *p* < 0.01.

## Data Availability

The original contributions presented in the study are included in the article/supplementary material, further inquiries can be directed to the corresponding author.

## Supplementary Materials

The following supporting information can be downloaded at https://www.mdpi.com/article/10.3390/foods13071033/s1. Table S1: Operating chromatographic conditions, MS setting of UHPLC-Q-Orbitrap-MS system, and Lipidsearch^TM^ parameters. Table S2: Deuterated lipids in QC samples. Table S3: Details on the individual lipids identified. Figure S1: Qualitative lipid fingerprint of mozzarella samples with the number of lipids related to each lipid subclass. Lipids were identified in positive and negative acquisition modes. Figure S2: Box plots of lipid subclass areas related to solvent mixtures. Figure S3: Relative differences in extraction capability of lipid subclasses using ethanol/ethyl acetate (blue) and IPA/ethyl acetate (red) compared to methanol/ethyl acetate.
