# Supplementary material for: Microwave-Assisted Extraction/UHPLC-Q-Orbitrap-MS-Based Lipidomic Workflow for Comprehensive Study of Lipids in Soft Cheese
Source: Foods. 2024 Mar 28;13(7):1033. doi: 10.3390/foods13071033 (PMC11012187; doi:10.3390/foods13071033)
Supplement: Supplementary file 1 [file foods-13-01033-s001.zip › foods-2883464-supplementary.pdf]

Supplementary Material

Microwave-Assisted Extraction/UHPLC-Q-Orbitrap-MS-Based Lipidomic Workflow for Comprehensive Study of Lipids in Soft Cheese

M. Campaniello, V. Nardelli, R. Zianni*, M. Tomaiuolo, O. Miedico, M. Iammarino, A. Mentana

Istituto Zooprofilattico Sperimentale della Puglia e della Basilicata, Via Manfredonia, 20-71121 Foggia, Italy

*** Corresponding author.**

E-mail address: rosalia.zianni@izspb.it

Table S1

Operating chromatographic conditions, MS setting of UHPLC-Q-Orbitrap-MS system and Lipidsearch™ parameters.

**Operating chromatographic conditions**				
**Sample temperature**:<br>18 °C	**Column and security guard column:**<br>Accucore C30 column (150 x 2.1 mm 2.6 µm column, Thermo) and ULTRA Cartridges UHPLC wide-pore C18 (AJ0-8769, 2 × 4.6 mm ID, with sub-2 m particles, Phenomenex)	**Injection Volume**:<br>2 µL (positive ion mode);<br>4 µL (negative ion mode)	**Phase A:**<br>ACN/H_2O (60:40, v/v),<br>10 mM NH_4HCO_2 and 0.1% HCO_2H<br>**Phase B:**<br>IPA/ACN (90:10, v/v),<br>10 mM NH_4HCO_2 and 0.1% HCO_2H<br>(Flow rate: 270 µL min^{-1})	**Elution gradient:**<br>**Time** (min) **/ B** (%)**:**<br>0/25-4.0/43-4.1/55-12.0/65-18.0/85-20.0/100-26.0/100-26.5/30-28.0/25-32.5/25

**MS setting**				
**Scan range**:<br>150-2000 m/z	**Full scan resolution** (FWHM): 70,000<br>**Multiple data-dependent (dd-MS^2) scan resolution** (FWHM): 17,500	**Spray voltage**:<br>3.4 kV (positive ion mode); 3.3 kV (negative ion mode)	**Capillary temperature**: 290 °C<br>**Auxiliary gas heater**: 290 °C	**AGC Target:** 1e6<br><br>**AGC target for dd-MS^2:** 2e5

**Data processing<br>Lipidsearch™ software:**			
**Search Parameters:**<br>**Class**: ALL Lipids<br>**Ions:**<br>+ H; $+NH_4$; +Na; $+(CH_3CH_2)_3NH$; $+(CH_3)_2NH_2$; $+H-H_2O$; $+H-2H_2O$; +2H (+)<br><br>-H; +HCOO; $+CH_3COO$; -2H; $-CH_3$ (-)	**Identification:**<br>Precursor tolerance in ppm: 5.0 (+); 8.0 (-)<br>Product tolerance in ppm: 8.0 (+); 10.0 (-)<br>m-Score threshold: 5.0<br>Database: General; HCD; Oxid. GPL; labelled GPL, GL, SP, ChE	**Search Filters:**<br>Top rank filter: on<br>Main node filter: all isomer peak<br>FA priority<br>ID quality filter: A, B, C, D	**Alignment Parameters:**<br>Search Type: Product<br>Normalize type: None<br>Alignment method: Mean<br>R.T. Tolerance: 0.1<br>Calculate unassigned peak area: on<br>Top rank filter: on<br>Main node filter: Main isomer peak<br>m-score Threshold: 5.0<br>c-score Threshold: 2.0<br>ID Quality filter: A, B, C, D

Table S2

Deuterated lipids in QC samples

LipidMolec	Calc Mass	BaseRt	Mean Peak Area	RSD %
Negative				
LPC(18:1D7)	528.3921	4.706	6.00E+07	1.157
LPE(18:1D7)	486.3451	4.753	3.93E+07	2.560
PC(18:1D7_15:0)	752.6061	14.112	3.37E+07	1.081
PE(18:1D7_15:0)	710.5591	14.126	1.96E+07	3.954
PG(18:1D7_15:0)	741.5537	12.667	7.00E+07	3.154
PI(18:1D7_15:0)	829.5698	12.203	2.87E+07	6.067
SM(d36:2D9)	737.6397	12.949	2.87E+07	5.446
Positive				
Cer(m32:0)	495.5015	13.457	9.31E+07	2.820
ChE(18:1D7)	657.6441	9.667	3.95E+08	15.959
LPC(18:1D7)	528.3921	4.651	2.75E+08	3.193
LPE(18:1D7)	486.3451	4.34	5.39E+07	2.542
PC(33:1D7)	752.6061	14.105	3.99E+08	3.146
PE(18:1D7_15:0)	710.5591	14.157	6.41E+07	3.476
PG(18:1D7_15:0)	741.5537	12.672	8.75E+07	2.137
PI(18:1D7_15:0)	829.5698	12.212	3.10E+07	4.054
SM(d36:2D9)	737.6397	12.934	2.00E+08	6.039
TG(15:0_18:1D7_15:0)	811.7646	23.036	7.36E+07	3.334

Table S3

Details on the individual lipids identified

LipidMolec	Calc Mass	MainIon
TG(10:0_10:0_10:0)	554.4546	$+NH_4$
TG(10:0_10:0_12:0)	582.4859	$+NH_4$
TG(10:0_10:0_12:1)	580.4703	$+NH_4$
TG(10:0_12:0_12:0)	610.5172	$+NH_4$
TG(10:0_12:0_14:0)	638.5485	$+NH_4$
TG(10:0_14:0_18:1)	720.6268	$+NH_4$
TG(10:0_17:1_18:1)	760.6581	$+NH_4$
TG(10:0_18:1_18:2)	772.6581	$+NH_4$
TG(10:0_18:2_18:2)	770.6424	$+NH_4$
TG(11:0_18:1_18:2)	786.6737	$+NH_4$
TG(12:0_14:0_14:0)	694.6111	$+NH_4$
TG(12:0_18:2_18:2)	798.6737	$+NH_4$
TG(12:0_18:2_18:3)	796.6581	$+NH_4$
TG(15:0_10:0_12:0)	652.5642	$+NH_4$
TG(15:0_10:0_14:0)	680.5955	$+NH_4$
TG(15:0_10:0_16:0)	708.6268	$+NH_4$
TG(15:0_10:0_18:1)	734.6424	$+NH_4$
TG(15:0_10:1_18:1)	732.6268	$+NH_4$
TG(15:0_10:1_18:2)	730.6111	$+NH_4$
TG(15:0_12:0_14:0)	708.6268	$+NH_4$
TG(15:0_12:0_18:1)	762.6737	+Na
TG(15:0_14:0_14:0)	736.6581	$+NH_4$
TG(15:0_14:0_16:0)	764.6894	$+NH_4$
TG(15:0_14:0_18:1)	790.705	+Na
TG(15:0_14:1_18:1)	788.6894	$+NH_4$
TG(15:0_16:0_16:0)	792.7207	$+NH_4$
TG(15:0_16:0_18:1)	818.7363	$+NH_4$
TG(15:0_16:0_18:2)	816.7207	+Na
TG(15:0_16:0_20:4)	840.7207	$+NH_4$
TG(15:0_18:1_18:1)	844.752	$+NH_4$
TG(15:0_18:1_18:2)	842.7363	$+NH_4$
TG(15:0_18:1_18:3)	840.7207	$+NH_4$
TG(15:0_6:0_10:0)	568.4703	$+NH_4$
TG(15:0_6:0_14:0)	624.5329	$+NH_4$
TG(15:0_6:0_16:0)	652.5642	$+NH_4$
TG(15:0_6:0_16:1)	650.5485	$+NH_4$
TG(15:0_6:0_18:1)	678.5798	$+NH_4$
TG(15:0_6:0_8:0)	540.439	$+NH_4$
TG(15:0_8:0_14:0)	652.5642	$+NH_4$
TG(15:0_8:0_16:1)	678.5798	$+NH_4$

TG(16:0_10:0_14:0)	694.6111	$+NH_4$
TG(16:0_10:0_17:1)	734.6424	$+NH_4$
TG(16:0_10:1_18:3)	742.6111	$+NH_4$
TG(16:0_12:0_14:0)	722.6424	$+NH_4$
TG(16:0_12:0_20:4)	798.6737	$+NH_4$
TG(16:0_14:0_14:0)	750.6737	$+NH_4$
TG(16:0_14:0_16:0)	778.705	$+NH_4$
TG(16:0_14:0_17:1)	790.705	$+NH_4$
TG(16:0_14:0_18:1)	804.7207	+Na
TG(16:0_14:0_20:4)	826.705	$+NH_4$
TG(16:0_14:0_20:5)	824.6894	$+NH_4$
TG(16:0_16:0_16:0)	806.7363	$+NH_4$
TG(16:0_16:0_17:1)	818.7363	$+NH_4$
TG(16:0_16:0_18:1)	832.752	+Na
TG(16:0_16:0_20:5)	852.7207	$+NH_4$
TG(16:0_16:1_18:1)	830.7363	+Na
TG(16:0_16:1_20:5)	850.705	$+NH_4$
TG(16:0_17:0_18:1)	846.7676	+Na
TG(16:0_17:1_18:1)	844.752	$+NH_4$
TG(16:0_17:1_18:2)	842.7363	$+NH_4$
TG(16:0_18:1_18:1)	858.7676	+Na
TG(16:0_18:1_20:5)	878.7363	$+NH_4$
TG(16:0_18:1_21:0)	902.8302	$+NH_4$
TG(16:0_18:1_22:0)	916.8459	$+NH_4$
TG(16:0_18:1_23:0)	930.8615	$+NH_4$
TG(16:0_18:1_23:1)	928.8459	$+NH_4$
TG(16:0_18:1_24:0)	944.8772	$+NH_4$
TG(16:0_18:1_24:1)	942.8615	$+NH_4$
TG(16:0_18:2_18:2)	854.7363	$+NH_4$
TG(16:0_6:0_10:0)	582.4859	$+NH_4$
TG(16:0_6:0_14:0)	638.5485	$+NH_4$
TG(16:0_6:0_16:0)	666.5798	$+NH_4$
TG(16:0_6:0_17:0)	680.5955	$+NH_4$
TG(16:0_6:0_18:1)	692.5955	$+NH_4$
TG(16:0_6:0_20:4)	714.5798	$+NH_4$
TG(16:0_6:0_6:0)	526.4233	$+NH_4$
TG(16:0_8:0_10:0)	610.5172	$+NH_4$
TG(16:1_10:0_12:0)	664.5642	$+NH_4$
TG(16:1_10:0_18:1)	746.6424	$+NH_4$
TG(16:1_12:0_18:1)	774.6737	$+NH_4$
TG(16:1_14:1_18:1)	800.6894	$+NH_4$
TG(16:1_18:1_18:1)	856.752	$+NH_4$
TG(16:1_18:1_18:2)	854.7363	$+NH_4$
TG(16:1_18:1_18:3)	852.7207	$+NH_4$
TG(16:1_18:2_18:2)	852.7207	$+NH_4$

TG(16:1_18:2_18:3)	850.705	$+NH_4$
TG(16:1_6:0_18:1)	690.5798	$+NH_4$
TG(16:1_6:0_18:2)	688.5642	$+NH_4$
TG(16:1_6:0_18:3)	686.5485	$+NH_4$
TG(16:1_6:0_6:0)	524.4077	$+NH_4$
TG(16:1_8:0_18:2)	716.5955	$+NH_4$
TG(17:0_18:1_18:1)	872.7833	$+NH_4$
TG(17:0_18:1_18:2)	870.7676	$+NH_4$
TG(17:0_18:1_18:3)	868.752	$+NH_4$
TG(17:0_6:0_18:1)	706.6111	$+NH_4$
TG(18:0_15:0_16:0)	820.752	$+NH_4$
TG(18:0_16:0_16:0)	834.7676	$+NH_4$
TG(18:0_16:0_17:0)	848.7833	$+NH_4$
TG(18:0_16:0_17:1)	846.7676	$+NH_4$
TG(18:0_16:0_18:0)	862.7989	+Na
TG(18:0_16:0_18:1)	860.7833	$+NH_4$
TG(18:0_16:0_20:0)	890.8302	$+NH_4$
TG(18:0_16:0_21:0)	904.8459	$+NH_4$
TG(18:0_16:0_22:0)	918.8615	$+NH_4$
TG(18:0_16:0_23:0)	932.8772	$+NH_4$
TG(18:0_16:0_24:0)	946.8928	$+NH_4$
TG(18:0_17:0_18:0)	876.8146	$+NH_4$
TG(18:0_17:0_18:1)	874.7989	$+NH_4$
TG(18:0_17:1_18:0)	874.7989	$+NH_4$
TG(18:0_17:1_18:1)	872.7833	$+NH_4$
TG(18:0_17:1_18:2)	870.7676	$+NH_4$
TG(18:0_18:0_18:0)	890.8302	$+NH_4$
TG(18:0_18:0_18:1)	888.8146	$+NH_4$
TG(18:0_18:0_19:0)	904.8459	$+NH_4$
TG(18:0_18:0_20:0)	918.8615	$+NH_4$
TG(18:0_18:0_21:0)	932.8772	$+NH_4$
TG(18:0_18:0_22:1)	944.8772	$+NH_4$
TG(18:0_18:0_22:4)	938.8302	$+NH_4$
TG(18:0_18:0_23:0)	960.9085	$+NH_4$
TG(18:0_18:1_18:1)	886.7989	$+NH_4$
TG(18:0_18:1_19:0)	902.8302	$+NH_4$
TG(18:0_18:1_20:0)	916.8459	$+NH_4$
TG(18:0_18:1_20:1)	914.8302	$+NH_4$
TG(18:0_18:1_22:4)	936.8146	$+NH_4$
TG(18:0_18:1_22:5)	934.7989	$+NH_4$
TG(18:0_18:1_23:0)	958.8928	$+NH_4$
TG(18:0_18:1_23:1)	956.8772	$+NH_4$
TG(18:0_18:1_24:0)	972.9085	$+NH_4$
TG(18:0_18:1_24:1)	970.8928	$+NH_4$
TG(18:0_20:0_23:1)	986.9241	$+NH_4$

TG(18:0_6:0_16:0)	694.6111	$+NH_4$
TG(18:0_6:0_18:0)	722.6424	$+NH_4$
TG(18:0_6:0_18:1)	720.6268	$+NH_4$
TG(18:0_6:0_18:3)	716.5955	$+NH_4$
TG(18:1_10:1_18:3)	768.6268	$+NH_4$
TG(18:1_12:0_14:0)	748.6581	$+NH_4$
TG(18:1_12:0_17:1)	788.6894	$+NH_4$
TG(18:1_12:0_18:1)	802.705	$+NH_4$
TG(18:1_14:0_14:0)	776.6894	$+NH_4$
TG(18:1_14:0_17:1)	816.7207	$+NH_4$
TG(18:1_14:0_18:1)	830.7363	+Na
TG(18:1_14:1_17:1)	814.705	$+NH_4$
TG(18:1_14:1_18:1)	828.7207	$+NH_4$
TG(18:1_14:1_18:2)	826.705	$+NH_4$
TG(18:1_14:1_18:3)	824.6894	$+NH_4$
TG(18:1_17:1_18:1)	870.7676	$+NH_4$
TG(18:1_17:1_18:2)	868.752	$+NH_4$
TG(18:1_18:1_18:1)	884.7833	$+NH_4$
TG(18:1_18:1_18:2)	882.7676	$+NH_4$
TG(18:1_18:1_20:3)	908.7833	$+NH_4$
TG(18:1_18:1_21:0)	928.8459	$+NH_4$
TG(18:1_18:1_21:1)	926.8302	$+NH_4$
TG(18:1_18:1_22:1)	940.8459	$+NH_4$
TG(18:1_18:1_22:5)	932.7833	$+NH_4$
TG(18:1_18:1_23:1)	954.8615	$+NH_4$
TG(18:1_18:1_24:1)	968.8772	$+NH_4$
TG(18:1_18:2_18:2)	880.752	$+NH_4$
TG(18:1_18:2_18:3)	878.7363	$+NH_4$
TG(18:1_18:2_20:3)	906.7676	$+NH_4$
TG(18:1_18:2_20:4)	904.752	$+NH_4$
TG(19:0_18:1_18:1)	900.8146	$+NH_4$
TG(19:0_18:1_18:2)	898.7989	$+NH_4$
TG(19:1_18:0_18:0)	902.8302	$+NH_4$
TG(19:1_18:0_18:1)	900.8146	$+NH_4$
TG(19:1_18:1_18:1)	898.7989	$+NH_4$
TG(19:1_18:1_18:2)	896.7833	$+NH_4$
TG(20:0_16:0_17:0)	876.8146	$+NH_4$
TG(20:0_16:0_18:1)	888.8146	$+NH_4$
TG(20:0_18:1_18:2)	912.8146	$+NH_4$
TG(20:1_18:1_18:1)	912.8146	$+NH_4$
TG(20:1_18:1_18:2)	910.7989	$+NH_4$
TG(25:0_16:0_18:0)	960.9085	$+NH_4$
TG(25:0_16:0_18:1)	958.8928	$+NH_4$
TG(25:0_18:0_18:1)	986.9241	$+NH_4$
TG(25:0_18:1_18:1)	984.9085	$+NH_4$

TG(25:1_16:0_16:0)	930.8615	$+NH_4$
TG(25:1_16:0_18:1)	956.8772	$+NH_4$
TG(26:0_16:0_18:0)	974.9241	$+NH_4$
TG(26:0_18:0_18:1)	1000.9398	$+NH_4$
TG(26:0_18:1_18:1)	998.9241	$+NH_4$
TG(26:0_18:1_18:2)	996.9085	$+NH_4$
TG(4:0_10:0_10:0)	470.3607	$+NH_4$
TG(4:0_10:0_11:0)	484.3764	$+NH_4$
TG(4:0_10:0_12:0)	498.392	+Na
TG(4:0_10:0_16:0)	554.4546	$+NH_4$
TG(4:0_10:0_18:0)	582.4859	$+NH_4$
TG(4:0_10:0_20:4)	602.4546	$+NH_4$
TG(4:0_10:1_12:1)	494.3607	$+NH_4$
TG(4:0_10:1_15:0)	538.4233	$+NH_4$
TG(4:0_10:1_16:0)	552.439	$+NH_4$
TG(4:0_10:1_18:0)	580.4703	$+NH_4$
TG(4:0_10:1_18:1)	578.4546	$+NH_4$
TG(4:0_10:1_18:2)	576.439	$+NH_4$
TG(4:0_10:1_18:3)	574.4233	$+NH_4$
TG(4:0_11:0_18:2)	592.4703	$+NH_4$
TG(4:0_11:1_14:1)	536.4077	$+NH_4$
TG(4:0_11:1_16:0)	566.4546	$+NH_4$
TG(4:0_12:0_18:3)	604.4703	$+NH_4$
TG(4:0_12:0_20:4)	630.4859	$+NH_4$
TG(4:0_12:1_18:1)	606.4859	$+NH_4$
TG(4:0_12:2_16:0)	578.4546	$+NH_4$
TG(4:0_13:0_18:2)	620.5016	$+NH_4$
TG(4:0_14:0_14:0)	582.4859	$+NH_4$
TG(4:0_14:0_15:0)	596.5016	$+NH_4$
TG(4:0_14:0_16:0)	610.5172	$+NH_4$
TG(4:0_14:0_18:1)	636.5329	$+NH_4$
TG(4:0_14:0_18:2)	634.5172	$+NH_4$
TG(4:0_14:0_18:3)	632.5016	$+NH_4$
TG(4:0_14:0_20:4)	658.5172	$+NH_4$
TG(4:0_14:0_22:5)	684.5329	$+NH_4$
TG(4:0_14:1_18:3)	630.4859	$+NH_4$
TG(4:0_14:3_16:0)	604.4703	$+NH_4$
TG(4:0_15:0_16:0)	624.5329	$+NH_4$
TG(4:0_15:0_16:1)	622.5172	$+NH_4$
TG(4:0_15:0_18:1)	650.5485	$+NH_4$
TG(4:0_15:0_18:2)	648.5329	$+NH_4$
TG(4:0_15:0_18:3)	646.5172	$+NH_4$
TG(4:0_16:0_16:0)	638.5485	$+NH_4$
TG(4:0_16:0_16:1)	636.5329	$+NH_4$
TG(4:0_16:0_17:0)	652.5642	$+NH_4$

TG(4:0_16:0_17:1)	650.5485	$+NH_4$
TG(4:0_16:0_18:0)	666.5798	$+NH_4$
TG(4:0_16:0_18:2)	662.5485	$+NH_4$
TG(4:0_16:0_18:3)	660.5329	$+NH_4$
TG(4:0_16:0_19:1)	678.5798	$+NH_4$
TG(4:0_16:0_20:4)	686.5485	$+NH_4$
TG(4:0_16:0_20:5)	684.5329	$+NH_4$
TG(4:0_16:1_18:3)	658.5172	$+NH_4$
TG(4:0_17:0_18:0)	680.5955	$+NH_4$
TG(4:0_17:0_18:1)	678.5798	$+NH_4$
TG(4:0_17:0_18:2)	676.5642	$+NH_4$
TG(4:0_17:1_18:0)	678.5798	$+NH_4$
TG(4:0_17:1_18:1)	676.5642	$+NH_4$
TG(4:0_17:1_18:2)	674.5485	$+NH_4$
TG(4:0_18:0_18:0)	694.6111	$+NH_4$
TG(4:0_18:0_18:1)	692.5955	$+NH_4$
TG(4:0_18:0_18:3)	688.5642	$+NH_4$
TG(4:0_18:1_18:1)	690.5798	$+NH_4$
TG(4:0_18:1_18:2)	688.5642	$+NH_4$
TG(4:0_18:1_19:1)	704.5955	$+NH_4$
TG(4:0_18:1_20:4)	712.5642	$+NH_4$
TG(4:0_18:1_20:5)	710.5485	$+NH_4$
TG(4:0_18:2_18:2)	686.5485	$+NH_4$
TG(4:0_18:2_18:3)	684.5329	$+NH_4$
TG(4:0_6:0_10:0)	414.2981	$+NH_4$
TG(4:0_6:0_12:0)	442.3294	$+NH_4$
TG(4:0_6:0_13:0)	456.3451	$+NH_4$
TG(4:0_6:0_14:0)	470.3607	$+NH_4$
TG(4:0_6:0_14:1)	468.3451	+Na
TG(4:0_6:0_15:0)	484.3764	$+NH_4$
TG(4:0_6:0_16:0)	498.392	+Na
TG(4:0_6:0_16:1)	496.3764	+Na
TG(4:0_6:0_17:1)	510.392	$+NH_4$
TG(4:0_6:0_18:0)	526.4233	$+NH_4$
TG(4:0_6:0_18:1)	524.4077	$+NH_4$
TG(4:0_6:0_18:2)	522.392	$+NH_4$
TG(4:0_6:0_18:3)	520.3764	$+NH_4$
TG(4:0_6:0_20:4)	546.392	$+NH_4$
TG(4:0_8:0_12:0)	470.3607	$+NH_4$
TG(4:0_8:0_12:1)	468.3451	$+NH_4$
TG(4:0_8:0_14:0)	498.392	+Na
TG(4:0_8:0_14:2)	494.3607	$+NH_4$
TG(4:0_8:0_16:0)	526.4233	$+NH_4$
TG(4:0_8:0_17:1)	538.4233	$+NH_4$
TG(4:0_8:0_18:1)	552.439	$+NH_4$

TG(4:0_8:0_20:4)	574.4233	$+NH_4$
TG(4:0_9:0_14:1)	510.392	$+NH_4$
TG(4:0_9:0_18:1)	566.4546	$+NH_4$
TG(4:0_9:0_18:2)	564.439	$+NH_4$
TG(6:0_10:0_10:1)	496.3764	$+NH_4$
TG(6:0_10:0_12:0)	526.4233	$+NH_4$
TG(6:0_10:0_14:0)	554.4546	$+NH_4$
TG(6:0_10:0_14:1)	552.439	$+NH_4$
TG(6:0_10:0_17:1)	594.4859	$+NH_4$
TG(6:0_10:0_18:1)	608.5016	$+NH_4$
TG(6:0_10:0_18:2)	606.4859	$+NH_4$
TG(6:0_10:0_18:3)	604.4703	$+NH_4$
TG(6:0_10:0_20:4)	630.4859	$+NH_4$
TG(6:0_12:0_14:0)	582.4859	$+NH_4$
TG(6:0_12:0_20:4)	658.5172	$+NH_4$
TG(6:0_14:0_14:0)	610.5172	$+NH_4$
TG(6:0_14:0_18:1)	664.5642	$+NH_4$
TG(6:0_14:0_18:2)	662.5485	$+NH_4$
TG(6:0_14:1_17:1)	648.5329	$+NH_4$
TG(6:0_14:1_18:1)	662.5485	$+NH_4$
TG(6:0_14:1_18:2)	660.5329	$+NH_4$
TG(6:0_14:1_18:3)	658.5172	$+NH_4$
TG(6:0_17:1_18:1)	704.5955	$+NH_4$
TG(6:0_17:1_18:2)	702.5798	$+NH_4$
TG(6:0_18:1_18:1)	718.6111	$+NH_4$
TG(6:0_18:1_18:2)	716.5955	$+NH_4$
TG(6:0_18:1_20:4)	740.5955	$+NH_4$
TG(6:0_18:1_20:5)	738.5798	$+NH_4$
TG(6:0_18:2_18:2)	714.5798	$+NH_4$
TG(6:0_18:2_18:3)	712.5642	$+NH_4$
TG(6:0_6:0_10:0)	442.3294	$+NH_4$
TG(6:0_6:0_12:0)	470.3607	$+NH_4$
TG(6:0_6:0_14:0)	498.392	$+NH_4$
TG(6:0_6:0_18:2)	550.4233	$+NH_4$
TG(6:0_6:0_18:3)	548.4077	$+NH_4$
TG(6:0_8:0_10:0)	470.3607	$+NH_4$
TG(6:0_8:0_10:1)	468.3451	$+NH_4$
TG(6:0_8:0_12:0)	498.392	+Na
TG(6:0_8:0_12:1)	496.3764	$+NH_4$
TG(6:0_8:0_14:0)	526.4233	$+NH_4$
TG(6:0_8:0_14:1)	524.4077	$+NH_4$
TG(6:0_8:0_17:1)	566.4546	$+NH_4$
TG(6:0_8:0_18:1)	580.4703	$+NH_4$
TG(6:0_8:0_18:2)	578.4546	$+NH_4$
TG(6:0_8:0_18:3)	576.439	$+NH_4$

TG(6:0_8:0_20:4)	602.4546	$+NH_4$
TG(6:0_9:0_12:0)	512.4077	$+NH_4$
TG(8:0_10:0_10:0)	526.4233	$+NH_4$
TG(8:0_10:0_18:1)	636.5329	$+NH_4$
TG(8:0_10:0_18:2)	634.5172	$+NH_4$
TG(8:0_10:0_18:3)	632.5016	$+NH_4$
TG(8:0_18:1_18:2)	744.6268	$+NH_4$
TG(8:0_18:1_18:3)	742.6111	$+NH_4$
TG(8:0_8:0_8:0)	470.3607	$+NH_4$
TG(9:0_18:1_18:2)	758.6424	$+NH_4$
TG(9:0_9:0_10:0)	526.4233	$+NH_4$
TG(18:1+O_14:0_16:0)	820.7156	$+NH_4$
TG(18:1+O_14:0_18:0)	848.7469	$+NH_4$
TG(18:1+O_14:0_18:1)	846.7313	$+NH_4$
TG(18:1+O_16:0_16:0)	848.7469	$+NH_4$
TG(18:1+O_16:0_18:0)	876.7782	$+NH_4$
TG(18:1+O_16:0_18:1)	874.7626	$+NH_4$
TG(18:1+O_18:0_18:0)	904.8095	$+NH_4$
TG(18:1+O_18:0_18:1)	902.7939	$+NH_4$
TG(18:1+O_18:1_18:1)	900.7782	$+NH_4$
TG(18:2+O_16:0_16:0)	846.7313	$+NH_4$
TG(18:2+O_16:0_18:0)	874.7626	$+NH_4$
TG(18:2+O_16:0_18:1)	872.7469	$+NH_4$
TG(18:2+O_18:0_18:1)	900.7782	$+NH_4$
TG(18:2+OO_15:0_16:0)	848.7105	+Na
TG(18:2+OO_16:0_17:0)	876.7418	+Na
TG(18:2+OO_16:0_17:1)	874.7262	+Na
TG(18:2+OO_16:0_19:0)	904.7731	+Na
TG(18:2+OO_16:0_21:0)	932.8044	+Na
TG(18:2+OO_16:0_21:1)	930.7888	+Na
TG(18:2+OO_17:0_18:3)	898.7262	+H
TG(18:2+OO_17:1_18:3)	896.7105	+H
TG(18:2+OO_18:3_19:0)	926.7575	+H
TG(18:2+OO_18:3_21:0)	954.7888	+H
TG(18:2+OO_18:3_21:1)	952.7731	+H
DG(10:0_12:0)	428.3502	+Na
DG(10:0_14:0)	456.3815	+Na
DG(10:0_14:1)	454.3658	+Na
DG(10:0_18:1)	510.4284	+Na
DG(10:0_18:2)	508.4128	$+NH_4$
DG(10:0_18:3)	506.3971	$+NH_4$
DG(12:0_14:0)	484.4128	+Na
DG(12:0_18:2)	536.4441	$+NH_4$
DG(14:0_18:2)	564.4754	$+NH_4$

DG(14:0_18:3)	562.4597	$+NH_4$
DG(16:0_10:0)	484.4128	+Na
DG(16:0_12:0)	512.4441	+Na
DG(16:0_14:0)	540.4754	+Na
DG(16:0_14:1)	538.4597	$+NH_4$
DG(16:0_16:0)	568.5067	+Na
DG(16:0_18:1)	594.5223	$+NH_4$
DG(16:0_18:2)	592.5067	$+NH_4$
DG(16:0_6:0)	428.3502	+Na
DG(16:0_8:0)	456.3815	+Na
DG(16:1_18:1)	592.5067	$+NH_4$
DG(18:0_16:0)	596.538	$+NH_4$
DG(18:0_18:0)	624.5693	$+NH_4$
DG(18:0_18:1)	622.5536	$+NH_4$
DG(18:0_18:2)	620.538	$+NH_4$
DG(18:1_14:0)	566.491	+Na
DG(18:1_18:1)	620.538	$+NH_4$
DG(18:1_18:2)	618.5223	$+NH_4$
DG(18:1_18:3)	616.5067	$+NH_4$
DG(4:0_14:0)	372.2876	$+NH_4$
DG(4:0_16:0)	400.3189	$+NH_4$
DG(4:0_18:0)	428.3502	$+NH_4$
DG(4:0_18:1)	426.3345	+Na
DG(4:0_18:2)	424.3189	$+NH_4$
DG(6:0_14:0)	400.3189	$+NH_4$
DG(6:0_18:1)	454.3658	+Na
DG(6:0_18:2)	452.3502	$+NH_4$
DG(8:0_14:0)	428.3502	$+NH_4$
DG(8:0_18:1)	482.3971	+Na
DG(8:0_18:2)	480.3815	$+NH_4$
DG(18:2+OO_9:0)	526.387	$+NH_4$
Cer(d16:1_22:0)	593.5747	+HCOO
Cer(d16:1_23:0)	607.5903	+HCOO
Cer(d16:1_24:0)	621.606	+HCOO
Cer(d18:1_16:0)	537.5121	+HCOO
Cer(d18:1_23:0)	635.6216	+HCOO
Cer(d18:1_24:0)	649.6373	+HCOO
Hex1Cer(d18:1_16:0)	699.5649	+HCOO
Hex1Cer(d18:1_22:0)	783.6588	+HCOO
Hex1Cer(d18:1_23:0)	797.6745	+HCOO
Hex1Cer(d18:1_24:0)	811.6901	+HCOO
Hex2Cer(d18:1_22:0)	945.7116	+HCOO
Hex2Cer(d18:1_23:0)	959.7273	+HCOO
Hex2Cer(d18:1_24:0)	973.7429	+HCOO
LPC(12:0)	439.2699	+H

LPC(14:0)	467.3012	+H
LPC(15:0)	481.3168	+H
LPC(16:0)	495.3325	+H
LPC(16:1)	493.3168	+H
LPC(18:0)	523.3638	+H
LPC(18:1)	521.3481	+H
LPC(18:2)	519.3325	+H
LPE(16:0)	453.2855	-H
LPE(18:0)	481.3168	-H
LPE(18:1)	479.3012	-H
LPE(18:2)	477.2855	-H
LPI(18:0)	600.3275	-H
LPS(18:0)	525.3067	-H
LPS(18:1)	523.291	-H
PC(16:0_14:0)	705.5309	+HCOO
PC(16:0_16:0)	733.5622	+HCOO
PC(16:0_18:1)	759.5778	+HCOO
PC(16:0_18:2)	757.5622	+HCOO
PC(18:0_18:1)	787.6091	+HCOO
PC(18:1_18:1)	785.5935	+HCOO
PC(28:0)	677.4996	+H
PC(32:1)	731.5465	+H
PC(36:3)	783.5778	+H
PE(16:0_16:1)	689.4996	+H
PE(16:0_18:1)	717.5309	+H
PE(16:0_18:2)	715.5152	+H
PE(16:1_18:1)	715.5152	+H
PE(18:0_18:1)	745.5622	+H
PE(18:0_18:2)	743.5465	+H
PE(18:1_18:1)	743.5465	+H
PE(18:1_18:2)	741.5309	+H
PI(16:0_18:1)	836.5415	-H
PI(18:0_18:1)	864.5728	-H
PI(18:0_18:2)	862.5571	-H
PI(18:0_20:4)	886.5571	-H
PI(18:1_18:1)	862.5571	-H
PS(18:0_18:1)	789.552	-H
PS(18:0_18:2)	787.5363	-H
PS(38:4)	811.5363	-H
SM(d32:1)	674.5363	+HCOO
SM(d34:1)	702.5676	+HCOO
SM(d38:1)	758.6302	+HCOO
SM(d39:0)	774.6615	+HCOO
SM(d39:1)	772.6458	+HCOO
SM(d40:1)	786.6615	+HCOO

SM(d41:1)	800.6771	+HCOO
SM(d42:1)	814.6928	+HCOO
BisMePA(16:0_14:0)	648.473	+Na
ChE	386.3549	$+H-H_2O$

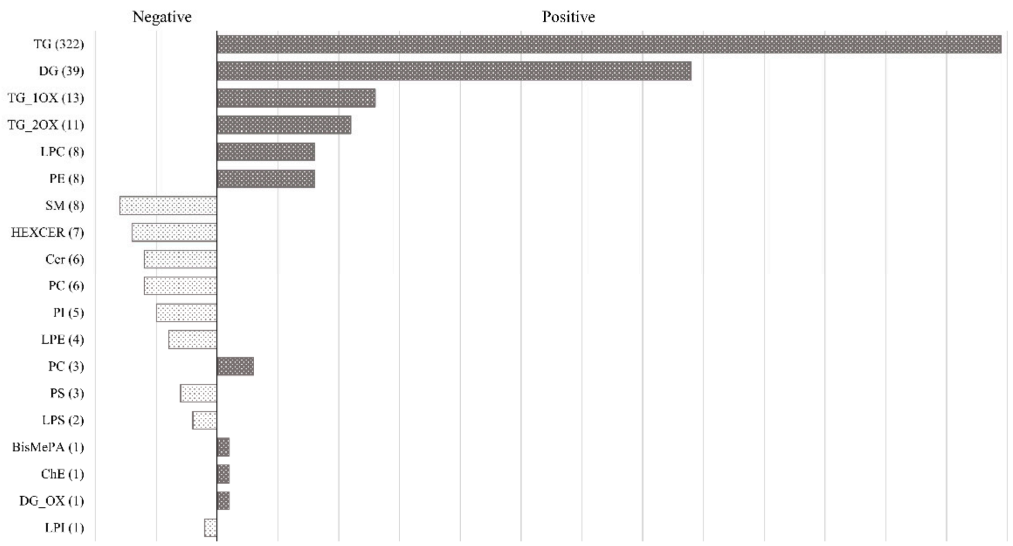

Figure S1. Qualitative lipid fingerprint of mozzarella samples with the number of lipids related to each lipid subclass. Lipids were identified in positive and negative acquisition modes. For better graphical visualization, TG line is shown as 322/5.

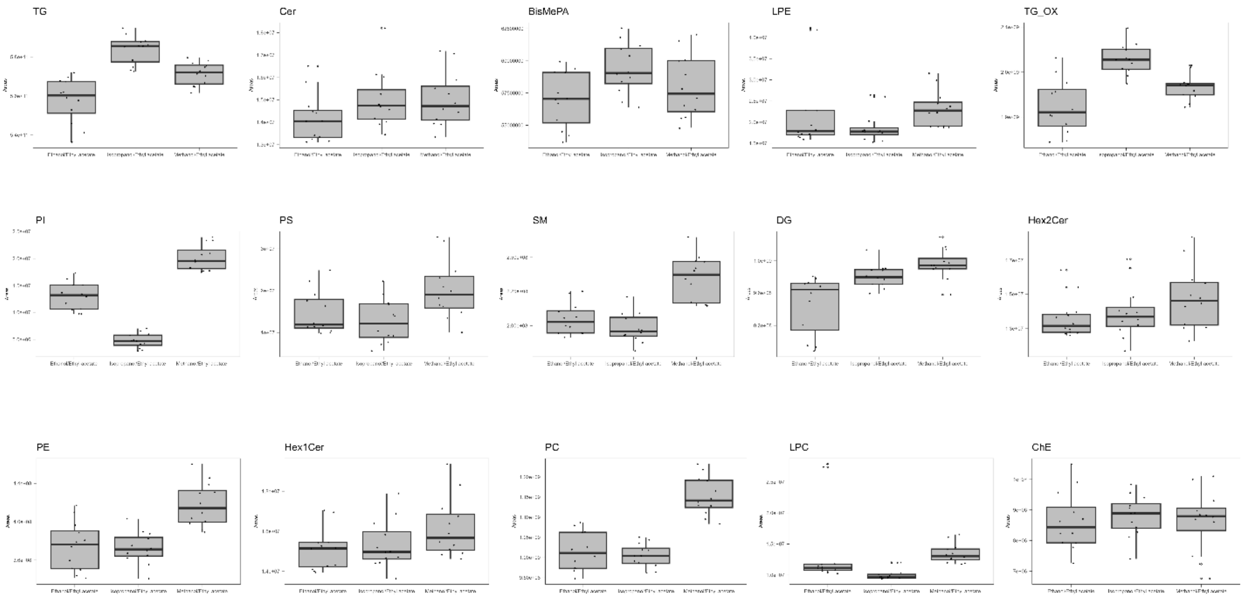

Figure S2. Box Plots of lipid subclass areas related to solvent mixtures.

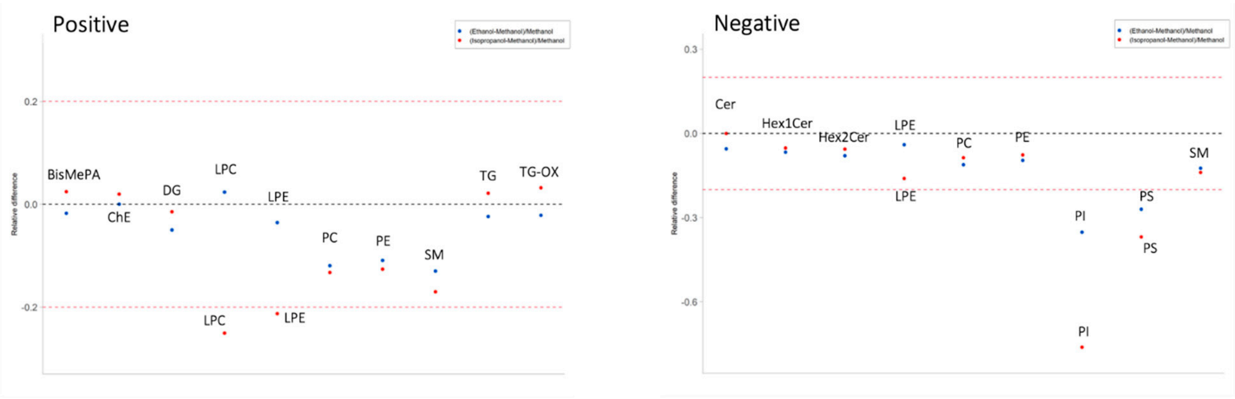

Figure S3. Relative differences of extraction capability of lipid subclasses using, ethanol/ethyl acetate (blue) and isopropanol/ethyl acetate (red) compared to methanol/ethyl acetate.
